# Dynamic Analysis of Stakeholders' Decision‐Making in Power Battery Recycling Considering Risks

**DOI:** 10.1002/gch2.202500313

**Published:** 2025-09-24

**Authors:** Juan Huang, Zhe Wang, Zhenggang He, Weiwei Xu, Feng Luo

**Affiliations:** ^1^ School of Transportation and Logistics Southwest Jiaotong University Chengdu 610031 China; ^2^ School of Economics and Management Sichuan Tourism University Chengdu 610100 China

**Keywords:** CVaR, decision‐making, evolutionary game, power battery recycling, risk‐induced loss

## Abstract

The management of risks in retired battery recycling chains remains a challenge, with a particular scarcity of dynamic models that can quantify risk loss and its impact on the co‐evolution of stakeholders' strategies. To address this gap, a quantitative model integrating network calculus with conditional value‐at‐risk (CVaR) is proposed to quantify risk‐induced loss. This study explores how varying risk parameters affect stakeholders' revenues, risk‐induced loss, and their strategic behaviors. The results indicate that the impact of risk on government and recyclers differs based on their strategies, showing heightened sensitivity to risk under negative strategies. Moreover, the influence of risk parameters on risk‐induced loss changes over time, with risk management capabilities peaking at a 64.1% contribution rate before declining. Government decision‐making exhibits volatility in low‐risk scenarios, leading to fluctuations in consumers' behaviors. Risks play a pivotal role in propelling sustainable development in the recycling market. While stakeholders initially lean toward negative strategies when risk‐induced losses are minimal, escalating losses prompt a reassessment of such approaches, increasing the likelihood of transitioning to positive strategies. These findings provide valuable insights for enhancing risk management in power battery recycling.

## Graphical abstract

The risk‐induced loss quantification model based on conditional value‐at‐risk (CVaR) is established to elucidate the impact of various risk parameters on stakeholders' revenues and evolutionary paths, as well as identify the key parameters that affect risk‐induced loss. The findings offer insights for risk management in power battery recycling.

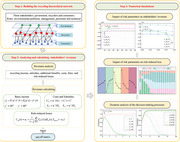

## 1 Introduction

With the increasing attention to global sustainable development and environmental protection, developing the new energy vehicle (NEV) industry has become one of the crucial avenues to reduce automotive exhaust emissions, mitigate energy crises, and promote the upgrading of the automotive industry.^[^ ^1^, ^2^ ^]^ However, the rapid increase in retired power batteries poses severe challenges to the present society. Inappropriately handling a large amount of retired power batteries can cause leakage of carcinogenic and mutagenic substances, thereby seriously polluting the environment.^[^ ^3^ ^]^ Additionally, the closed‐loop recycling of positive electrode materials for power batteries is significant for ensuring the supply of key metals (Li, Ni, Co, and Mn) and meeting the demand for environmental sustainability.^[^ ^4^ ^]^ The recycling of retired power batteries is a recognized green production method with environmental, economic, and social benefits, and it is also an essential link in green supply chain management.^[^ ^5^, ^6^ ^]^

Governments around the world have introduced a series of policies and plans to promote the sustainable development of the NEV industry and the power battery recycling industry.^[^ ^7^, ^8^, ^9^, ^10^, ^11^ ^]^ For example, the RoHS restrictions on hazardous substances force recyclers to optimize hazardous substance separation technologies and improve the recovery rate of high‐purity materials. The new EU Battery Regulation (2023) pertains to mandatory recycling, carbon footprint, and full lifecycle traceability; such strict requirements are conducive to promoting supply chain transparency and facilitating closed‐loop material circulation.^[^ ^12^ ^]^ Nevertheless, due to technical barriers, high investment costs, lack of government support policies, and other reasons, the current recycling rate of retired power batteries is far from satisfactory, with a significant proportion of power batteries still needing to be properly managed.^[^ ^13^ ^]^

Stakeholders involved in power battery recycling are showing a trend of diversified development, covering all aspects of the power battery production chain (including government, manufacturers, remanufacturers, retailers, professional recyclers, third‐party recycling platforms, and consumers). The initial stage of recycling operations involves the interaction between consumers and recyclers.^[^ ^14^ ^]^ The government plays a pivotal role in advancing battery recycling. Key factors influencing the behavior and effectiveness of participants in the recycling process include subsidy policies, regulatory oversight, deposit return systems, carbon taxes, and other external mechanisms.^[^ ^15^, ^16^ ^]^ Particularly, establishing rational reward and punishment mechanisms enables the government to maximize social welfare at the lowest cost.^[^ ^17^, ^18^, ^19^ ^]^ Meanwhile, as a provider of recycling services, the recyclers play a crucial role in the recycling system. It has been clarified that the recycling channel, recycling price, enterprise strategy, and environmental performance of the enterprise will directly affect the recovery rate of retired power batteries.^[^ ^20^ ^]^ As the provider of retired power batteries, consumers play an important role in determining the flow direction of retired batteries. It is essential to highlight that the active involvement of various recycling stakeholders is crucial for perpetuating the optimization and effectiveness of recycling systems. Moreover, the choices made by individual stakeholders have the potential to significantly impact the entire recycling process. Therefore, the research on the synergy effect of recycling stakeholders from a multi‐stakeholder perspective has been widely studied in many papers.

However, risk research is of great significance for the recycling of retired power batteries.^[^ ^21^ ^]^ The intricate recycling processes of power batteries, coupled with the intricate relationships among diverse recycling stakeholders and the inherent toxicity of the batteries, give rise to various risks.^[^ ^13^ ^]^ These risks encompass potential disruptions in recycling channels, failures in treatment technologies, environmental accidents, shifts in policies, plummeting metal prices, fire outbreaks, and leaks of hazardous substances.^[^ ^22^ ^]^ Furthermore, these risks have the capacity to propagate, disseminate, and accumulate among stakeholders, ultimately leading to disruptions in Closed‐Loop Supply Chains (CLSCs) and influencing the decision‐making processes of individual stakeholders, thereby impacting the efficiency of power battery recycling. Evidently, a research gap exists concerning the influence of risks on the dynamic evolution mechanism of recycling entities. These motivate us to focus on investigating the decision‐making processes of recycling stakeholders with a consideration of risks. We view the behaviors of government, recyclers, and consumers as a dynamic process of mutual co‐evolution and interaction. Employing an evolutionary game approach, the study aims to capture the interactions and decision‐making processes among stakeholders through the lens of risk. The study addresses the following research questions:

1. How do risk parameters impact the revenues of the government, recyclers, and consumers?
2. What are the primary factors influencing risk‐induced losses?
3. What is the dynamic evolution and co‐evolution mechanism of policy choices among the government, recyclers, and consumers?

To address these inquiries, a hierarchical “government‐recycler‐consumer” recycling network is constructed, integrating network calculus with conditional value at risk (CVaR) to develop a model for quantifying risk‐induced losses. Through this model, the paper explores the mechanisms by which risk influences the revenue of each stakeholder and the evolution of stakeholder behaviors. The contributions of this work are as follows:

1. A theoretical framework focused on risk has been established, incorporating the concept of risk‐driven dynamic evolution into the power battery recycling system. This novel approach provides a fresh perspective on the dynamics of multi‐agent interactions.
2. A quantification model of risk‐induced loss has been developed, combining conditional value‐at‐risk (CVaR) with the network calculus method, addressing the difficulties associated with accurately evaluating risk‐induced loss.
3. The impact of risk parameters on stakeholders' revenues, risk‐induced loss, and evolution of stakeholders' decision‐making is revealed, and the underlying driving force of risk‐driven market transition to sustainable development is elucidated.
4. The application of the Taguchi technique enhances the systematic and robust nature of the study, enabling the identification of key factors influencing risk‐induced losses and their respective significance levels. This represents a novel endeavor to integrate engineering methodologies into the field of decision science.

The paper is organized as follows: Section 2 provides a literature review, Section 3 outlines the methodology, Section 4 presents the numerical simulations, Section 5 discusses the results, and Section 6 offers conclusions and recommendations.

## 2 Literature Review

### 2.1 Recycling Decision‐Making

Research on recycling decision‐making typically focuses on supply chain perspectives, including closed‐loop supply chains and multi‐agent collaboration mechanisms. Studies also compare efficiency and profit distribution across various recycling models. To address the complex, multi‐objective, and uncertain decision‐making environment, Multiple Attribute or Multiple Criteria Decision Making (MADM/MCDM) is commonly employed to quantitatively assess the strengths and weaknesses of technical schemes.^[^ ^23^, ^24^, ^25^ ^]^ Furthermore, technologies such as blockchain and digital battery passports enhance traceability, trust, and data transparency in recycling processes.^[^ ^26^, ^27^, ^28^ ^]^ The closed‐loop supply chain serves as a fundamental framework for battery recycling decisions, with existing models often utilizing evolutionary games, Markov decision processes, Nash equilibria, and other approaches to investigate strategic choices, cooperative incentives, and dynamic evolution paths among different stakeholders.^[^ ^29^, ^30^, ^31^, ^32^ ^]^ For instance, Tang et al. design a reward‐penalty mechanism and policy, using Stackelberg game theory to analyze the impact on power battery recycling.^[^ ^33^ ^]^ Gao et al. analyze the strategy selection of the three participants in the process of power battery recycling by constructing a tripartite evolutionary game model.^[^ ^34^ ^]^ Moreover, Li et al. simulate the dynamic evolution process of each stakeholder strategy and analyze the impact of digital transformation factors on the evolution trend.^[^ ^35^ ^]^ In addition, Tian et al. utilized agent‐based modeling and geographic information systems to establish a model for a cross‐regional recycling system for power batteries. This model aimed to assess the effects of subregional differentiation policies on formal recycling rates, emission reductions, and financial efficiency.^[^ ^36^ ^]^ In a similar vein, Wang et al. developed a two‐layer heterogeneous network model to explore the coevolution dynamics between residents and recyclers in EV battery recycling, highlighting the role of policy interventions in promoting formal recycling strategies.^[^ ^14^ ^]^ However, their research primarily focuses on policy‐driven behavioral changes without explicitly incorporating risk parameters into the decision‐making framework. Moreover, while they acknowledge the time‐dependent nature of policy diffusion, they do not investigate how risk accumulation over time directly impacts revenues and strategic stability. While Du et al. have effectively employed evolutionary game theory to optimize government regulatory parameters, such as subsidies and fines, for curbing irregular recycling, the dynamic impact of endogenous risk parameters on stakeholder revenues and strategic evolution remains underexplored.^[^ ^37^ ^]^ Nie et al. developed a tripartite evolutionary game and system dynamics model to assess the effects of policy interventions and market factors on stakeholder strategy evolution.^[^ ^38^ ^]^ However, their analysis presumed stakeholder risk neutrality and overlooked risk preferences. In this study, we fill this gap by introducing a risk‐centered theoretical framework to evaluate how these intrinsic characteristics, rather than external policy mechanisms, influence stakeholder profits and evolutionary trajectories.

### 2.2 Risk of Battery Recycling

At present, some efforts have been dedicated to investigating the risk of recycling power batteries. In the past few years, Bayesian networks, fault tree analysis, fuzzy logic, gray degree of possibility, MCDM, and CVaR have been used for risk research of power batteries.^[^ ^39^, ^40^, ^41^, ^42^ ^]^ Introducing risk control measures in supply chain collaboration is essential for managing potential risks such as future decommissioned battery scale explosions, material price fluctuations, policy changes, and multi‐disaster impacts.^[^ ^43^ ^]^ Previous studies have incorporated risk factors into pricing strategies and coordination mechanisms for power batteries, recycling network design, and CLSC, leading to positive outcomes.^[^ ^44^, ^45^ ^]^ However, the significance of risks and uncertainties in the recovery process has not been adequately emphasized in game models. Additionally, the dynamic and time‐sensitive nature of risks in recycling management has been overlooked. Recent research has made significant strides in identifying and prioritizing risks within the LIB recycling supply chain. For instance, Afroozi et al. developed an integrated Gray Delphi‐DEMATEL‐ANP framework coupled with zero‐sum game theory to assess risks and determine optimal mitigation strategies, effectively highlighting critical risks such as environmental pollution and insufficient support programs.^[^ ^41^ ^]^ However, their study primarily focuses on the static prioritization of risk entities and a bilateral adversarial perspective, leaving the dynamic impact of risk parameters on multi‐stakeholder decision‐making and revenue structures less explored. Bhuyan et al. statically mapped the interrelationships among barriers to Li‐ion battery recycling using a multi‐stakeholder Grey‐DEMATEL approach.^[^ ^46^ ^]^ While their study identifies critical factors, it does not model their dynamic evolution or quantify their impact on economic outcomes. This research extends their work by introducing a dynamic framework to analyze how these identified risks, as time‐variant attributes, propagate and affect stakeholder revenues and strategic interactions over time.

## 3 Methodology

**Figure 1:**
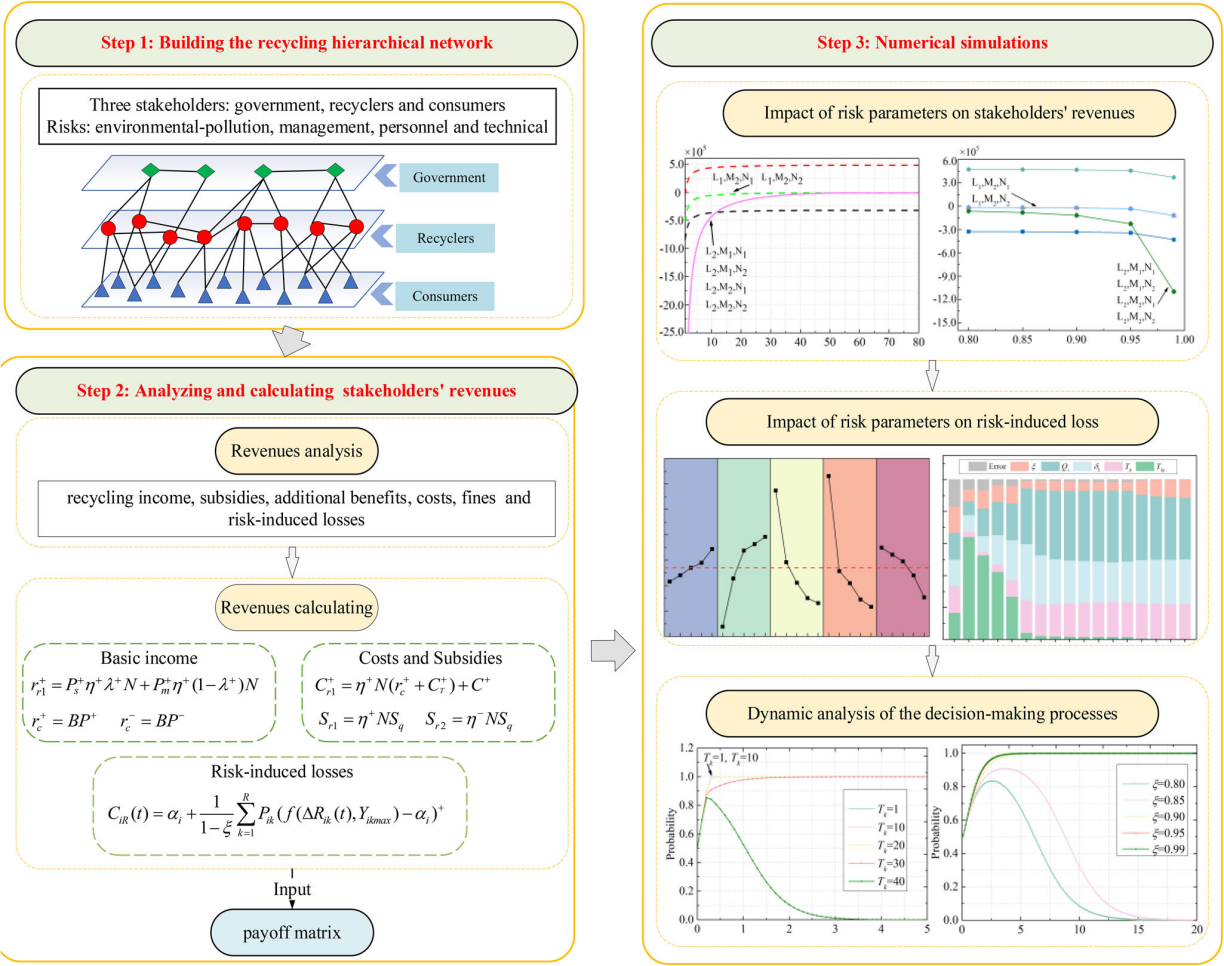
The framework of dynamic analysis.

We conducted a dynamic analysis of the decision‐making processes of governments, recyclers, and consumers from a risk perspective. As shown in **Figure** **1**, the study is divided into three phases: i) building the recycling hierarchical network, ii) analyzing and calculating stakeholders' revenues, and iii) dynamic analysis of the decision‐making processes. The initial phase involves stakeholder identification and risk assessment, emphasizing the analysis of interrelations to establish a hierarchical recycling network. Subsequently, the focus shifts to evaluating the composition of entities' revenues and estimating revenues by integrating network calculations with CVaR to quantify risk‐induced losses and integrate them into the payoff matrix. Lastly, the examination delves into assessing risk impacts on revenues and the decision‐making dynamics of entities across various scenarios.

### 3.1 Problem Description

**Figure 2:**
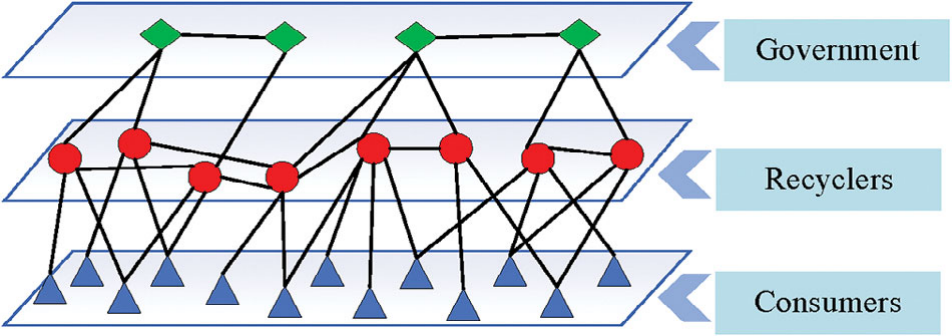
Government‐recyclers‐consumers recycling hierarchical network.

The energy vehicle battery recycling system is a complex system, involving diverse stakeholders engaged in dynamic strategic evolution and interactive interest relationships.^[^ ^14^ ^]^ This work constructs a recycling hierarchical network composed of governments, recyclers, and consumers. According to the hierarchical characteristics of recycling management, such a network is stratified into three levels. The number of nodes per layer is *N_K_*, where *K* = 1,2,3 represent the government layer, recycler layer, and consumer layer, respectively. The government is tasked with oversight and the establishment of policy standards, encompassing regulatory authorities from various administrative regions. Recyclers are responsible for providing recycling services, which include professional recycling companies, distributors, repair shops, and third‐party platforms engaged in recycling operations. Consumers are responsible for supplying used power batteries. A recycling network with power batteries as the link is formed among all levels, and a divergent multi‐level system is formed based on the government, as depicted in **Figure** **2**.

In such a recycling hierarchical network, the decisions of each stakeholder are closely related to their own responsibility and revenue. The government regulates the battery‐recycling system by setting standards, supervising compliance, and using subsidies or penalties to align recyclers and consumers with environmental goals, while the lack of supervision increases non‐compliance and systemic risk. The business goal of recyclers is usually to pursue a balance between economic benefits and environmental protection, so the different risks and benefits associated with compliant and non‐compliant behaviors have a significant influence on the decision‐making of recycling enterprises. Consumers select recycling channels according to price, convenience, and personal environmental awareness, and their choice of compliant recyclers can achieve environmental protection and social responsibility satisfaction, while avoiding potential legal risks.

**Figure 3:**
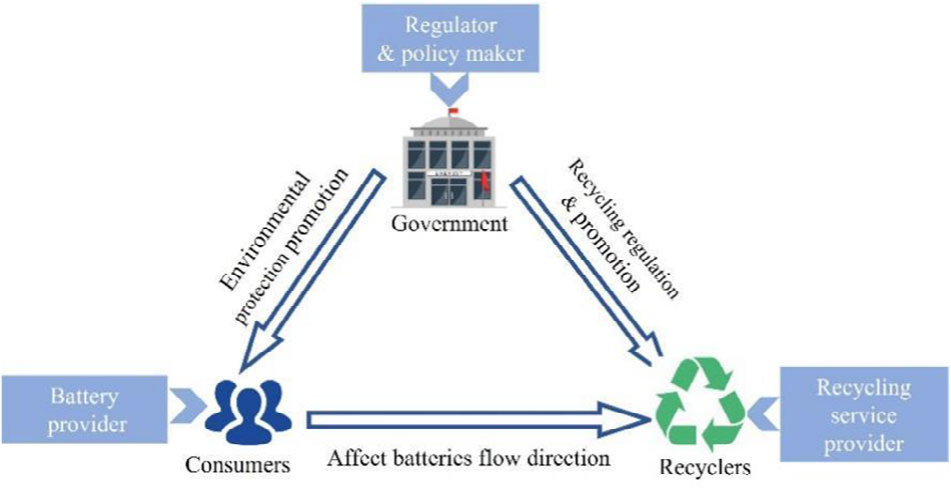
Stakeholders' evolutionary game relationships of the power battery recycling system.

Consequently, it is required for the government, recyclers, and consumers to engage in a holistic assessment of the risk and revenue associated with their decision‐making processes. The evolutionary game theory relationships of the three stakeholders within the recycling system are depicted in **Figure** **3**. It is worth noting that the CLSC of power batteries is affected by various risk factors, which stem from product design, manufacturing process, logistics process, market demand, and other aspects. As a dynamic and complex network, due to the involvement of a large number of consumers and recyclers, as well as frequent business transactions, risks flow upstream and downstream in the CLSC via medium (such as logistics, capital flow, and information flow). These risks persist in their propagation and aggregation through various stakeholders within the system. In scenarios where multiple stakeholders within the CLSC system fail to implement prompt mitigating actions to diminish or eradicate inherent risks, there exists a propensity for the emergence of a systemic crisis that can encompass the entire supply chain.

Aiming at solving this issue, Schoenherr has constructed a risk assessment index system from the aspects of production, cooperation, technology, benefit distribution, information transmission, seamless integration of forward and reverse supply chains, and environment to evaluate the risks of CLSC.^[^ ^47^ ^]^ To simplify the model for directly evaluating the impact of risks on stakeholder decisions, this work mainly considers four types of risks:

1. Environmental‐pollution risk: contamination of soil, water, and air by improper recycling activities.
2. Management risk: regulatory gaps that foster incomplete recycling systems, illegal dismantling, market disorder, and safety hazards.
3. Personnel risk: unsafe handling, incorrect battery classification, lack of protective measures, and insufficient safety awareness.
4. Technical risk: immature recycling technologies, equipment failure, and data‐security breaches.

Therefore, the core research question of this work is to identify and implement optimal behavioral strategies while considering the four types of risks, thereby fostering the sustainable growth of the power battery recycling and maximizing social welfare.

For the sake of convenience, the model parameters are defined in **Table** **1**.

**Table 1:** Parameter settings.

Parameters	
*N_K_*	Number of nodes per layer, where *K* = 1,2,3
$h_{g}^{r+}$, $h_{g}^{c+}$	Additional government revenue is generated by the active behavior of recyclers and consumers
$h_{r}^{r+}$	Additional recyclers' revenue generated by compliant recycling
*β*	Risk mitigation factor, 0 < *β* < 1
*C_Re_*, *C_Rm_*, *C_Rs_*, *C_Rt_*	Environmental pollution, management, personnel, and technical risk‐induced loss due to passive behavior
$C_{g}^{+}$, $C_{g}^{-}$	Cost of active and passive government regulation
$C_{c}^{+}$, $C_{c}^{-}$	Hassle the cost of consumers choosing compliant and non‐compliant recycling channels
$C_{r\;1}^{+}$, $C_{r\;2}^{+}$	Cost of compliant recyclers when consumer and recycler strategies are consistent and inconsistent
$C_{r\;1}^{-}$, $C_{r\;2}^{-}$	Cost of non‐compliant recyclers when consumer and recycler strategies are consistent and inconsistent
$r_{r\;1}^{+}$, $r_{r\;2}^{+}$	Basic revenue for compliant recyclers when consumer and recycler strategies are consistent and inconsistent
$r_{r\;1}^{-}$, $r_{r\;2}^{-}$	Basic revenue for non‐compliant recyclers when consumer and recycler strategies are consistent and inconsistent
$r_{c}^{+}$, $r_{c}^{-}$	Consumers' revenue for compliant and non‐compliant recycling channels
*f*	Punishments imposed on recyclers for non‐compliant recycling when the government actively regulates
$S_{r1}$, $S_{r2}$	Subsidies provided to recyclers for compliant recycling when consumer and recycler strategies are consistent and inconsistent
*N*	Number of tired batteries recycled by recyclers
*S_q_*	Subsidy amount per unit
*S_c_*	Rewards for consumers choosing formal recycling channels when the government actively regulates
$P_{s}^{+}$, $P_{m}^{+}$	Unit revenue from ladder utilization and dismantling for formal recyclers
$P_{s}^{-}$, $P_{m}^{-}$	Unit revenue from ladder utilization and dismantling for informal recyclers
*η* ^+^, *η* ^−^	Battery recycling rates corresponding to consistent and inconsistent consumer and recycler strategies
λ^+^, λ^−^	Ladder utilization rates of compliant and non‐compliant recyclers
$C_{T}^{+}$, $C_{T}^{-}$	Transportation and storage costs for compliant and non‐compliant recyclers
*P* ^+^, *P* ^−^	Recycling prices per unit of battery capacity for compliant and non‐compliant recyclers
*B*	Remaining capacity of retired batteries
*i*, *j*	Nodes in the network
*w_ij_*	Connectivity intensity between *i* and *j*, where *w_ij_* ∈ [0,100]
*H_Ki_*	Number of edges connecting node *i* at layer *K* to the nodes in the subsequent layer
*Q_i_*	Risk intensity
*R_ik_*	Risk faced by node *i*, *k* = 1, 2, …, *R*, where *R* = 4
*T_k_*	Risk period
*Y_ikmax_*	Risk threshold that node *i* can tolerate for *R_ik_*
*T_kc_*	risk management capability
*P_k_*	Average probability of encountering risk *k*
*C_iR_*	Average loss caused by risks for node *i*
*ξ*	Confidence level
*δ*_1_, *δ*_2_	Penalty coefficients for low and high risk, respectively, where *δ*_1_ << *δ*_2_
*α_i_*	VaR of node *i*

### 3.2 Model Assumption

Assumption 1: The government, recyclers, and consumers are all bounded rational stakeholders with learning abilities. The game participants engage in a recursive process of error analysis and learning to inform decision‐making, seeking the best strategy under the principle of revenue maximization, and constantly adjusting their decisions.

Assumption 2: The strategy set of the government L = {active regulation (L_1_), passive regulation (L_2_)}, with a probability vector *x* = (*x*, 1‐*x*). The strategy set of recyclers M = {compliant behavior (M_1_), non‐compliant behavior (M_2_)}, with a probability vector *y* = (*y*, 1‐*y*). The strategy set of consumers N = {formal recycling channels (N_1_), informal recycling channels (N_2_)}, the probability vector is *z* = (*z*, 1‐*z*). Here, *x*, *y*, *z* ∈ [0,1]. Active behavior refers to L_1_, M_1_, and N_1_, while passive behavior refers to L_2_, M_2_, and N_2_.

Assumption 3: Stakeholders face risks under both active and passive behaviors. However, the magnitude and nature of risk‐induced losses differ significantly. Active behaviors significantly mitigate, but do not eliminate, the magnitude of certain risk‐induced losses compared to passive behaviors. Specifically, we introduce a risk mitigation factor *β* (0 < *β* < 1) to quantify the risk reduction effect of active behavior. Under active behavior, effective risk‐induced loss is modeled as the product of *β* and total risk‐induced loss under passive behavior.

Assumption 4: Environmental pollution is caused solely by improper handling by recyclers. According to the polluter pays principle, the losses caused by environmental pollution shall be borne by the polluters.

### 3.3 Stakeholders' Revenues Analysis

#### 3.3.1 Recyclers' Revenue

The revenue of recyclers comprises basic recycling income, government subsidies, and additional benefits from compliant recycling, minus costs of purchasing and transportation, fines, and risk‐induced losses. Recyclers handle retired batteries through ladder utilization and dismantling. The alignment of consumer and recycler strategies influences battery recycling rates. Let *η* ^+^ and *η* ^−^ denote the recycling rates corresponding to consistent and inconsistent consumer and recycler strategies, respectively. The basic recycling income $r_{r\;1}^{+}$, $r_{r\;2}^{+}$, $r_{r\;1}^{-}$, $r_{r\;2}^{-}$, and costs $C_{r\;1}^{+}$, $C_{r\;2}^{+}$, $C_{r\;1}^{-}$, $C_{r\;2}^{-}$ of compliant and non‐compliant recyclers in two scenarios are calculated by:

(1) $$r_{r1}^{+}=P_{s}^{+}\eta^{+}\lambda^{+}N+P_{m}^{+}\eta^{+}\left(1-\lambda^{+}\right)N$$

(2) $$r_{r2}^{+}=P_{s}^{+}\eta^{-}\lambda^{+}N+P_{m}^{+}\eta^{-}\left(1-\lambda^{+}\right)N$$

(3) $$r_{r1}^{-}=P_{s}^{-}\eta^{+}\lambda^{-}N+P_{m}^{-}\eta^{+}\left(1-\lambda^{-}\right)N$$

(4) $$r_{r2}^{-}=P_{s}^{-}\eta^{-}\lambda^{-}N+P_{m}^{-}\eta^{-}\left(1-\lambda^{-}\right)N$$

(5)

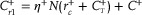



(6)

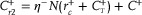



(7)

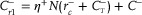



(8)

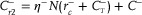

where *N* is the number of retired batteries recycled by recyclers. The unit revenues from ladder utilization and dismantling are denoted by $P_{s}^{+}$ and $P_{m}^{+}$ for formal recyclers, and $P_{s}^{-}$ and $P_{m}^{-}$ for informal recyclers, respectively. The ladder utilization rates of compliant and non‐compliant recyclers are represented by λ^+^ and λ^−^, respectively. Furthermore, 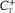 and 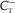 denote the transportation and storage costs, while *C* ^+^ and *C* ^−^represent fixed costs for compliant and non‐compliant recyclers. $r_{c}^{+}$ and $r_{c}^{-}$ represent the revenue from consumers for the compliant and non‐compliant recycling channels, respectively.

Furthermore, we calculate risk‐induced losses by combining network calculations with CVaR. In the framework of network calculus, nodes in the network are represented by *i* and *j*, and the connectivity intensity between them is represented by *w_ij_*, where *w_ij_* ∈ [0,100]. The larger *w_ij_* is, the stronger the connectivity intensity between nodes *i* and *j*. In particular, if there is no connection, then *w_ij_* = 0. The network calculus framework provides the foundational metrics for quantifying the exposure and criticality of each node within the battery recycling network. In this context, the connectivity intensity *w_ij_* is not merely a topological metric but is directly proportional to the volume of retired batteries or frequency of transactions flowing between nodes *i* and *j*.^[^ ^48^ ^]^ A higher *w_ij_* indicates that node *i* or *j* is a central hub in the material flow, handling larger quantities of potentially hazardous materials. Consequently, this heightened activity directly amplifies its exposure to various risks.^[^ ^49^ ^]^

Let *H_Ki_* denote the number of edges connecting node *i* at layer *K* to the nodes in the subsequent layer. The risk intensity *Q_i_*, derived from the network metrics *w_ij_* and *H_Ki_*, thus serves as a composite measure of a node's operational load and topological vulnerability. Its calculation is shown in Equation 9. A node with high *Q_i_* is not only processing a large volume of batteries but may also be critically dependent on max (*w_ij_*) and min(*w_ij_*). This makes it a prime candidate for risk accumulation. For instance, nodes with higher *Q_i_* values are more likely to handle a greater number of batteries, thereby elevating the risk of environmental hazards such as leakage, spillage, or improper storage.^[^ ^50^ ^]^ Conversely, nodes characterized by higher *H_Ki_* values exhibit intricate logistics and substantial transaction volumes, which can strain management systems and escalate risks such as documentation errors or non‐compliance. The heightened throughput exerts pressure on equipment and processes, potentially elevating failure rates during dismantling or cascading utilization, thereby introducing technical risks.^[^ ^51^ ^]^ Moreover, scaling up operations amplifies personnel workload and necessitates additional employee training, consequently augmenting the likelihood of personnel‐related risks.

(9) $$Q_{i}=\frac{\sum_{j=1}^{N_{K}}w_{ij}-\max\left(w_{ij}\right)}{\max\left(w_{ij}\right)-\min\left(w_{ij}\right)}+\frac{H_{Ki}-\max\left(H_{Ki}\right)}{\max\left(H_{Ki}\right)-\min\left(H_{Ki}\right)},i\neq j$$

Let *R_ik_* denote the risk faced by node *i*, *k* = 1, 2, …, *R*, where *R* = 4, representing environmental pollution risk, management risk, personnel risk, and technical risk, respectively. To investigate the variation of stakeholders' revenues to different risk parameters more clearly, we posit that all nodes are subject to an identical risk environment and exhibit uniform processing efficiency. And *T_k_* is the risk period. The value of *R_ik_* at time *t*, namely *R_ik_* (*t*), is calculated by:

(10) $$R_{ik}\left(t\right)=\frac{t}{T_{k}}Q_{i}+Q_{i}$$

The maximum cumulative value *Y_ikmax_* represents the risk threshold that node *i* can tolerate for *R_ik_*, which is determined by the following equation:

(11) $$Y_{ik\max}=T_{kc}\frac{Q_{i}}{T_{k}}+Q_{i}$$

where *T_kc_* is the risk management capability of node *i*. When *R_ik_* (*t*) surpasses *Y_ikmax_*, it could lead to the collapse of the entire recycling network or even the failure of recycling tasks.

The time‐dependent risk function *R_ik_* (*t*) therefore models how these inherent, static vulnerabilities evolve into dynamic risk states under the pressure of time. The threshold *Y_ikmax_* represents the node's risk‐bearing capacity, determined by its efficiency in mitigating.

However, the ultimate impact of these risks is not deterministic but stochastic, influenced by uncertainties in operational conditions, market fluctuations, and external events. The binary outcome of whether *R_ik_* (*t*) surpasses *Y_ikmax_* is an oversimplification for loss estimation. Therefore, we introduce the CVaR model and combine it with network calculus to construct a risk loss quantification model. CVaR quantifies the expected severity of losses in the worst‐case scenarios, which are precisely the scenarios where the risk states modeled by *R_ik_* (*t*) exceed the capacities *Y_ikmax_*.^[^ ^52^ ^]^ The risks faced by power battery recycling systems often exhibit the characteristics of “low frequency and high loss”, involving environmental responsibility, safety risks, and potentially huge compensation. Therefore, the decision‐makers typically require a more conservative risk assessment to avoid catastrophic consequences. Since the CVaR model shows significant advantages in quantifying downside risks, it is highly appropriate to utilize this model for conducting research on power battery recycling.^[^ ^53^, ^54^ ^]^ In addition, due to the excellent universality and risk management advantages, CVaR has been introduced into reverse logistics network design, supply chain, financial management, and integrated energy systems.^[^ ^55^, ^56^, ^57^, ^58^ ^]^

The mechanism of risk occurrence is complex. Statistical analysis of a large amount of historical data shows that the evolution of specific types of risk often conforms to certain distribution patterns.^[^ ^54^ ^]^ To simplify he model, we assume that *P_k_* is the average probability of node *i* encountering *R_ik_*. This approach focuses on the median trend of potential outcomes. The average loss caused by risks at time *t* when the confidence level is larger than *ξ* is represented by *C_iR_* (*t*), which is calculated by the following equations:

(12) $$\Delta R_{ik}\left(t\right)=R_{ik}\left(t\right)-Y_{ikmax}$$

(13) $$f\left(\Delta R_{ik}\left(t\right),Y_{ik\max}\right)=\left\{\begin{array}{cc} -\delta_{1}\Delta R_{ik}\left(t\right), & \Delta R_{ik}\left(t\right)\leq 0\\ \delta_{2}\Delta R_{ik}\left(t\right), & \Delta R_{ik}\left(t\right)>0 \end{array}\right.$$

(14) $$C_{iR}\left(t\right)=\alpha_{i}+\frac{1}{1-\xi }\sum_{k=1}^{R}P_{ik}{\left(f\left(\Delta R_{ik}\left(t\right),Y_{ik\max}\right)-\alpha_{i}\right)}^{+}$$

(15) $${\left(f\left(\Delta R_{ik}\left(t\right),Y_{ik\max}\right)-\alpha_{i}\right)}^{+}=\max\left\{f\left(\Delta R_{ik}\left(t\right),Y_{ik\max}\right)-\alpha_{i},0\right\}$$

where *ξ* reflects the level of risk aversion of decision‐makers, Δ*R_ik_* (*t*) is the difference between *R_ik_* and *Y_ikmax_* at time *t*, *δ*_1_ and *δ*_2_ are the penalty coefficients for low and high risk, respectively, and *δ*_1_ << *δ*_2_. *α_i_* is the Value‐at‐Risk (VaR) of node *i*.^[^ ^59^ ^]^

In essence, the CVaR provides a rigorous, time‐varying loss input to the payoff matrix. It translates the topological and operational vulnerabilities of the recycling network into a tangible economic disincentive within the strategic decision‐making process of each stakeholder. This integration allows us to simulate how stakeholders interact from a risk perspective.

#### 3.3.2 Government Revenue

Government revenue is determined by policy effectiveness, subtracting regulatory and incentive costs and losses due to management risks. Incentive costs include subsidies to recyclers *S_r_*_1_, *S_r_*_2_, and rewards to consumers *S_c_*. And *S_r_*_1_, *S_r_*_2_ are shown below:

(16) $$S_{r1}=\eta^{+}NS_{q}$$

(17) $$S_{r2}=\eta^{-}NS_{q}$$

where *S_q_* is the subsidy amount per unit.

#### 3.3.3 Consumers Revenue

Consumers revenue includes revenue from retired batteries and government subsidies *S_c_*, minus hassle costs. The revenue from retired batteries $r_{c}^{+}$, $r_{c}^{-}$ are shown below:

(18) $$r_{c}^{+}=BP^{+}$$

(19) $$r_{c}^{-}=BP^{-}$$

where *P* ^+^ and *P^—^* represent the recycling prices per unit of battery capacity for compliant and non‐compliant recyclers, respectively. *B* represents the remaining capacity of retired batteries.

Based on the above analysis, the tripartite evolutionary game payoff matrix for each stakeholder is shown in **Table** **2**, where *C_R_* = *C_Re_* +*C_Rm_* +*C_Rs_* +*C_Rt_*.

**Table 2:** The tripartite evolutionary game payoff matrix.

Strategies	Government	Recyclers	Consumers
(L_1_, M_1_, N_1_)	$h_{g}^{r+}+h_{g}^{c+}-C_{g}^{+}-S_{r1}-\eta^{+}NS_{c}-\beta C_{Rm}$	$r_{r1}^{+}+h_{r}^{r+}+S_{r1}-C_{r1}^{+}-\beta C_{R}$	$r_{c}^{+}+S_{c}-C_{c}^{+}$
(L_1_, M_1_, N_2_)	$h_{g}^{r+}-C_{g}^{+}-S_{r2}-\beta C_{Rm}$	$r_{r2}^{+}+h_{r}^{r+}+S_{r2}-C_{r2}^{+}-\beta C_{R}$	$r_{c}^{-}-C_{c}^{-}$
(L_1_, M_2_, N_1_)	$f-C_{g}^{+}-\beta C_{Rm}$	$r_{r2}^{-}-f-C_{R}-C_{r2}^{-}$	$r_{c}^{+}+S_{c}-C_{c}^{+}$
(L_1_, M_2_, N_2_)	$f-C_{g}^{+}-\beta C_{Rm}$	$r_{r1}^{-}-f-C_{R}-C_{r1}^{-}$	$r_{c}^{-}-C_{c}^{-}$
(L_2_, M_1_, N_1_)	$-C_{Rm}-C_{g}^{-}$	$r_{r1}^{+}+h_{r}^{r+}-C_{r1}^{+}-\beta C_{R}$	$r_{c}^{+}-C_{c}^{+}$
(L_2_, M_1_, N_2_)	$-C_{Rm}-C_{g}^{-}$	$r_{r2}^{+}+h_{r}^{r+}-C_{r2}^{+}-\beta C_{R}$	$r_{c}^{-}-C_{c}^{-}$
(L_2_, M_2_, N_1_)	$-C_{Rm}-C_{g}^{-}$	$r_{r2}^{-}-C_{R}-C_{r2}^{-}$	$r_{c}^{+}-C_{c}^{+}$
(L_2_, M_2_, N_2_)	$-C_{Rm}-C_{g}^{-}$	$r_{r1}^{-}-C_{R}-C_{r1}^{-}$	$r_{c}^{-}-C_{c}^{-}$

**Figure 4:**
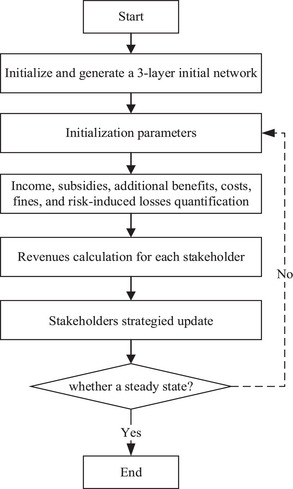
Flowchart of the evolutionary dynamics.

The flowchart of evolutionary dynamics is shown in **Figure** **4**. The workflow begins with the structure of the battery recycling network, which is quantified using network calculus. Initialization parameters are used to calculate the income, subsidies, additional benefits, costs, fines, and risk‐induced losses. And these calculated values are directly fed into the payoff functions of each stakeholder within the tripartite evolutionary game, where they act as a benefit or cost term, influencing strategic decision‐making. The resulting strategy dynamics can, over time, feed back and alter the network structure itself, creating a closed‐loop system.

## 4 Numerical Simulations

### 4.1 Parameter Settings

From the perspective of the reverse supply chain of power batteries, the key factors affecting stakeholder decision‐making are revenues. Here, computational analyses are conducted using MATLAB R2024a. The impact of risk on stakeholders' decision‐making is elucidated through equilibrium analysis and sensitivity analysis. Referring to the existing studies, we set *S_c_* = 2000 CNY/unit, *S_q_* = 1000 CNY/unit, *N* = 500, *f* = 10000 CNY, $P_{s}^{+}=23600$ CNY/unit, $P_{m}^{+}=6608$ CNY/unit, $P_{s}^{-}=33040$ CNY/unit, $P_{m}^{-}=9440$ CNY/unit, λ^+^ = 0.65, λ^−^ = 0.35.^[^ ^14^ ^]^ We use 80% of the battery capacity of the BYD Song as the sample, set *B* = 47.2kWh, *P* ^+^ = 100 CNY/kWh, $P^{-}=110$ CNY/kWh.^[^ ^14^, ^60^, ^61^ ^]^ The industry generally believes that the costs incurred by formal recycling companies in the transportation and storage of power batteries may be 5∼10 times higher than those incurred by informal channels. This paper assumes that the costs incurred by recyclers are equal to the transportation and storage costs, referring to existing research, setting 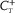 = 1154 CNY/unit, 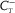 = 115 CNY/unit.^[^ ^62^ ^]^ In addition, we assume $h_{g}^{r+}$ = 600000 CNY, $h_{g}^{c+}$ = 600000 CNY, $C_{g}^{+}$ = 20000 CNY, $C_{g}^{-}$ = 1000 CNY, $h_{r}^{r+}$ = 850000 CNY, $C_{c}^{+}=200$ CNY, $C_{c}^{-}$ = 50 CNY, η^+^ = 1, η^−^ = 0.2, *C* ^+^ = 1600000 CNY, *C* ^−^ = 100000 CNY, and *β* = 0.1, which means that compliance can avoid 90% of potential risk‐induced losses.

To account for market competition, this paper sets the number of nodes *N_K_* = [4, 30, 500] in the recycling hierarchical network. Given the difficulty in acquiring precise risk parameters and industry‐specific data, assumptions were employed to establish risk parameters for enabling numerical simulation and theoretical examination of the model. Risks in nascent sectors like battery recycling were categorized as ranging from moderate to probable, leading to the assignment of risk occurrence probabilities *P_k_* within a rational interval of 0.05 to 0.25. Moreover, based on the cost‐ratio method, *δ*_1_: *δ*_2_ is set as 1:1000, and *δ*_1_ = 2. In addition, we set risk period *T_k_* = [40, 30, 20, 10], risk management capability *T_kc_* = [8, 10, 4, 5], *ξ* = 95%.^[^ ^63^ ^]^ The main parameter information and values are shown in **Table** **3**.

**Table 3:** Values of parameters.

Params.	Values	Unit	Params.	Values	Unit	Params.	Values	Unit
*S_c_*	2000	CNY/unit	η^+^	1	/	*C* ^+^	1600000	CNY
*S_q_*	1000	CNY/unit	η^−^	0.2	/	*C* ^−^	100000	CNY
*N*	500	unit	$C_{c}^{+}$	200	CNY	$h_{g}^{r+}$	600000	CNY
$P_{s}^{+}$	23600	CNY/unit	$C_{c}^{-}$	50	CNY	$h_{g}^{c+}$	600000	CNY
$P_{m}^{+}$	6608	CNY/unit	*f*	10000	CNY	$h_{r}^{r+}$	850000	CNY
$P_{s}^{-}$	33040	CNY/unit	*β*	0.1	/	$C_{g}^{+}$	20000	CNY
$P_{m}^{-}$	9440	CNY/unit	λ^+^	0.65	/	$C_{g}^{-}$	1000	CNY
*P* ^+^	100	CNY/kWh	λ^−^	0.35	/	*T_k_*	[40,30,20,10]	/
*P* ^–^	110	CNY/kWh	*ξ*	95	%	*T_kc_*	[8,10,4,5]	/
$C_{T}^{+}$	1154	CNY/unit	*δ*_1_	2	/	*P_k_*	[0.1, 0.2, 0.2, 0.25]	/
$C_{T}^{-}$	115	CNY/unit	*δ*_2_	2000	/	*N_K_*	[4,30,500]	/
*B*	47.2	kWh						

### 4.2 Stakeholders' Revenues

**Figure 5:**
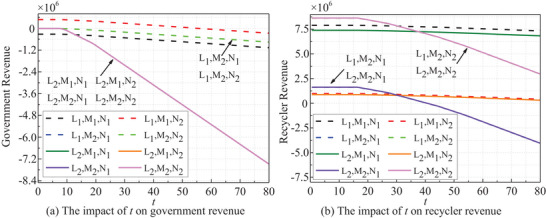
*t* dependent stakeholders' revenues.

The risk occurrence time *t*‐dependent revenues under different strategy combinations are displayed in **Figure** **5**. Government and recyclers' revenues remain stable during the initial phase of risk occurrence, then decline variably across strategy combinations, with the most significant decrease observed under passive regulation. Consumers' revenues remain stable throughout, with no significant direct impact from the timing of risk. Moreover, studies demonstrate that other risk parameters likewise have no direct impact on consumers' revenues. Thus, no further discussion about consumers will be provided in this section.

**Figure 6:**
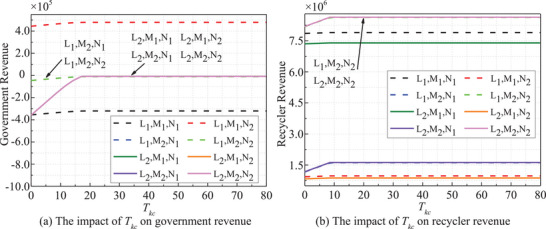
*T_kc_* dependent stakeholders' revenues.

**Figure** **6** shows the *T_kc_* dependent stakeholders' revenues (*t* = 20). Clearly, such variation trends are opposite to that of *t*. As *T_kc_* rises, government and recyclers' revenues increase across all strategies. Initially, earnings rise steady, stabilizing as *T_kc_* reaches a threshold. Notably, the stakeholders' revenues are more responsive to *T_kc_* under the passive strategies, exhibiting accelerated growth. The government prioritizes industry norms and public interests, while recyclers concentrate on enterprise profitability, resulting in divergent income levels and growth trajectories.

**Figure 7:**
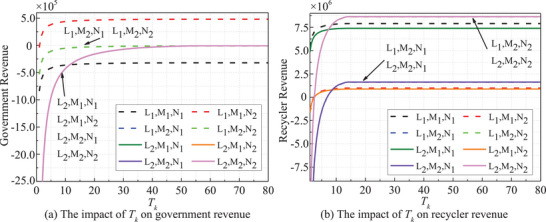
*T_k_* dependent stakeholders' revenues.

The risk period *T_k_*‐dependent stakeholders' revenues is shown in **Figure** **7** (*t* = 20). The revenues of both the government and recyclers show a notable increase with the duration of *T_k_* in the initial phase, eventually stabilizing in subsequent stages. A longer *T_k_* signifies a decrease in the frequency of risk events. Consequently, revenues for both entities tend to stabilize, particularly under an active strategy, which also results in a lower level influenced by *T_k_*.

**Figure 8:**
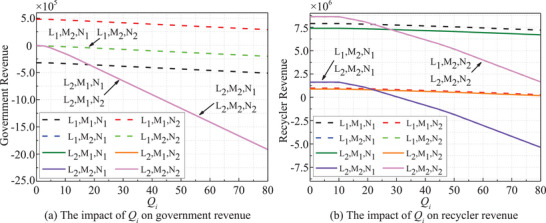
*Q_i_*‐dependent stakeholders' revenues.

**Figure 9:**
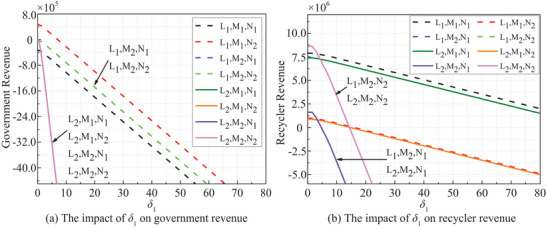
*δ*_1_ dependent stakeholders' revenues.

The impact of risk intensity *Q_i_* on revenues is evident in **Figure** **8** (*t* = 20). As *Q_i_* rises, both the government and recyclers' revenues decrease collectively. Revenues remain stable at low *Q_i_* levels but decline once *Q_i_* surpasses a certain threshold. Similarly, an escalation in the penalty coefficient *δ*_1_ adversely affects both the government and recyclers, as depicted in **Figure** **9**. The penalty coefficient plays a pivotal role in risk analysis, with a higher *δ*_1_ indicating stricter regulation or higher violation costs.

**Figure 10:**
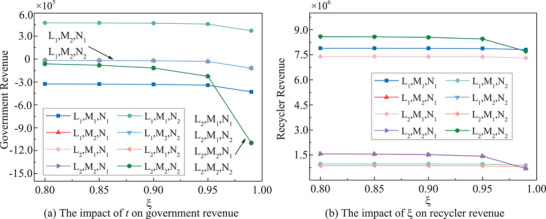
*ξ* dependent stakeholders' revenues.

In all strategies, both government and recyclers' revenues exhibit a notable decrease when ***ξ*** exceeds 0.95, as illustrated in **Figure** **10**. This decline is particularly pronounced under passive strategies, highlighting the critical threshold of 0.95 for decision‐making purposes.

### 4.3 Risk‐Induced Loss

The Taguchi technique is a powerful tool for parameter design. By using a special orthogonal table design, the entire parameter space can be studied within a limited number of experiments, which has been applied to various engineering problems.^[^ ^64^ ^]^ Here, based on the Taguchi technique, we evaluate the system stability under different parameter combinations by using Signal‐to‐Noise Ratio (SNR), thus reducing the impact of variation on the results. The L_25_(5^5^) orthogonal array is employed, with five control factors: risk management capability, risk period, penalty coefficient, risk intensity, and confidence level. Each factor has five levels, and the output parameter is risk‐induced loss, which is categorized as “lower is better” (*LB*). The SNR can be obtained by using Equation 20:^[^ ^65^ ^]^

(20) $$SNR=-10\;\log_{10}\left[\frac{1}{n}\sum_{i=1}^{n}y_{i}^{2}\right]$$

where *n* represents the number of repeated experiments, and *y* represents the observed value.

The levels for these factors were determined through sensitivity analysis in section 4.2. The lower bounds were set at values where the system behavior began to show a statistically significant/meaningful change from the baseline. The upper bounds were set at values beyond which the response variable plateaued, deteriorated significantly, or represented a practical operational limit. The five levels were then chosen to be equidistant within this empirically determined range. This approach ensures that our experimental design efficiently explores the entire region of interest where parameter variation has the most substantial effect, rather than wasting resources on regions with minimal impact. For instance, for risk management capability (Figure 6), the response curve showed a marked decline in revenue impact below a threshold of 17, beyond which the effect stabilized. Therefore, to capture the non‐linear behavior across the effective operational range, the lower and upper bounds were set at 1 and 20, respectively. Four intermediate levels (5, 10, 15) were then added at equal intervals to ensure a uniform exploration of this range. The same rationale was applied to define the levels of the remaining factors. This method ensures that the Taguchi experiment probes the parameter space where the most significant effects on performance are expected, enhancing the efficiency and relevance of the optimization process. The L_25_(5^5^) orthogonal array experimental design and SNR at *t* = 30 are shown in **Table** **4**.

**Table 4:** The L_25_(5^5^) experimental design of orthogonal array and SNR.

Exp. No.	Input Parameters					Calculated SNR of *C_CVaR_*, dB
	*T_kc_*	*T_k_*	*δ*_1_	*Q_i_*	*ξ*	
1	1	1	1	1	0.8	−89.5
2	1	10	15	25	0.85	−134.5
3	1	20	30	50	0.9	−144.1
4	1	30	45	75	0.95	−153.7
5	1	40	60	100	0.99	−170.2
6	5	1	15	50	0.95	−169.0
7	5	10	30	75	0.99	−172.5
8	5	20	45	100	0.8	−146.4
9	5	30	60	1	0.85	−102.3
10	5	40	1	25	0.9	−89.5
11	10	1	30	100	0.85	−169.5
12	10	10	45	1	0.9	−113.6
13	10	20	60	25	0.95	−146.9
14	10	30	1	50	0.99	−108.9
15	10	40	15	75	0.8	−126.1
16	15	1	45	25	0.99	−182.0
17	15	10	60	50	0.8	−144.5
18	15	20	1	75	0.85	−104.2
19	15	30	15	100	0.9	−134.7
20	15	40	30	1	0.95	−89.5
21	20	1	60	75	0.9	−170.6
22	20	10	1	100	0.95	−120.7
23	20	20	15	1	0.99	−89.5
24	20	30	30	25	0.8	−118.6
25	20	40	45	50	0.85	−128.6

**Figure 11:**
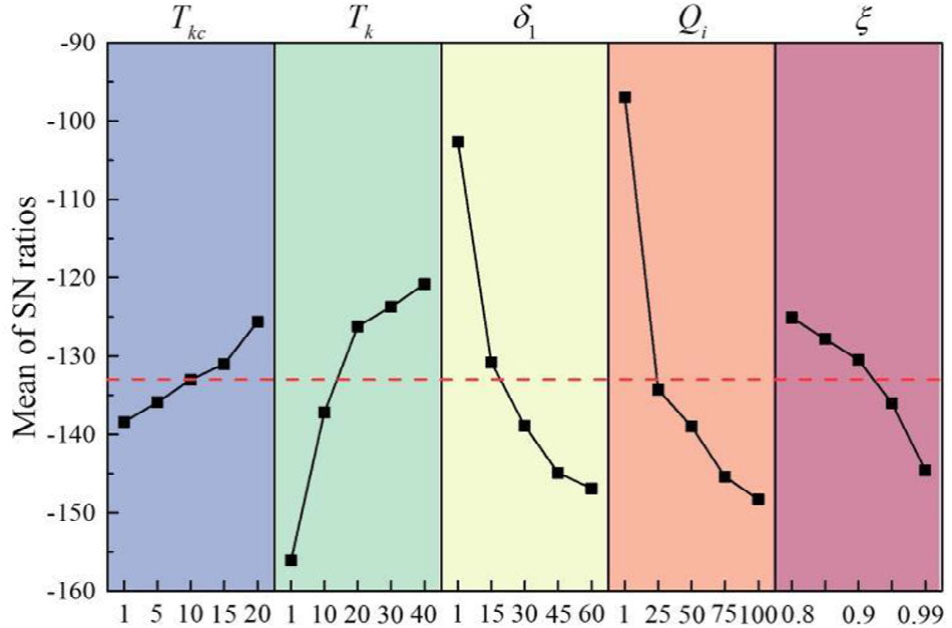
Main effects plot for SNR of risk‐induced loss.

To evaluate the significance level of risk parameters and their impact on risk‐induced loss, the SNR response table and analysis of variance (ANOVA) are employed. According to **Table** **5**, the contribution of each risk parameter to risk‐induced loss at *t* = 30, from high to low, is as follows: risk intensity (40.9% contribution), penalty coefficient (30.7% contribution), risk period (19.7% contribution), confidence level (5.7% contribution), and risk management capability (2.3% contribution). Four parameters show significance. Combining the main effects plot for SNR of risk‐induced loss (see **Figure** **11**), it is seen that when *t* = 30, *T_kc_* = 20 (Level 5), *T_k_* = 40 (Level 5), *δ*_1_ = 1 (Level 1), *Q_i_* = 1 (Level 1), and *ξ* = 0.80 (Level 1), the lowest risk‐induced loss is obtained, representing the optimal parameter configurations.

**Table 5:** Analysis of *C_CVaR_* results at *t* = 30.

Factor	Average SNR of *C_CVaR_*					SS	DF	MS	F	P	Contribution %
	1	2	3	4	5						
*T_kc_*	−138.4	−135.9	−133.0	−131.0	−125.6	483.3	4	120.8	3.0	0.160	2.3
*T_k_*	−156.1	−137.2	−126.2	−123.7	−120.8	4168.7	4	1042.2	25.5	0.004	19.7
*δ*_1_	−102.6	−130.8	−138.9	−144.9	−146.9	6496.4	4	1624.1	39.7	0.002	30.7
*Q_i_*	−96.9	−134.3	−139.0	−145.4	−148.3	8645.3	4	2161.3	52.8	0.001	40.9
*ξ*	−125.1	−127.8	−130.5	−136.0	−144.6	1200.7	4	300.2	7.3	0.040	5.7
Error						163.6	4	40.9			0.8
Total						21158.0	24				100.0

**Figure 12:**
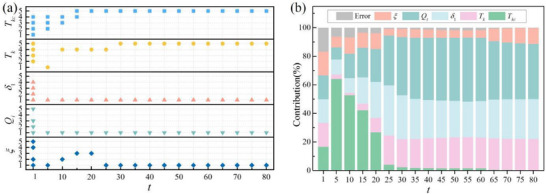
The risk occurrence time‐dependent optimal level of the factor and contribution rate.

In addition, the influence of time *t* on the optimal level and contribution rate of factors is analyzed (*t* ranges from 1 to 80 with an interval of five, see **Figure** **12**). As shown inFigure 12a, when *t* ≤ 15, the optimal level of risk management capability *T_kc_* fluctuated between 1 and 5 before stabilizing at level five, while the optimal level of *T_k_* also settled at level five. In contrast, risk intensity *Q_i_* and penalty coefficient *δ*_1_ remained consistently at level 1, while the confidence level *ξ* stabilized at level 1 after previous fluctuations. In terms of contribution rate (Figure 12b), that of *T_kc_* rose rapidly in the initial stage (reaching 64.1% at *t* = 5) and then gradually declined, suggesting a strong but transient early influence. Meanwhile, the contribution rates of *Q_i_*, *δ*_1_, and *T_k_* increased steadily over time and eventually stabilized, indicating cumulative or delayed effects. Notably, *Q_i_* maintained a high and stable contribution rate in the later phase (≈40%), confirming its role as a sustained dominant factor in risk‐induced loss. It is observed that the relatively large error at *t* = 1 likely resulted from the combined effects of initial transient dynamics, time‐varying parameter influences, and inherent limitations of the Taguchi method in handling initial non‐linearity and complexity. The subsequent rapid decrease and stabilization of error indicate that the system reached a robust state, underscoring the effectiveness of the Taguchi method in robustness optimization.

### 4.4 Evolution of Stakeholders' Decisions

**Figure 13:**
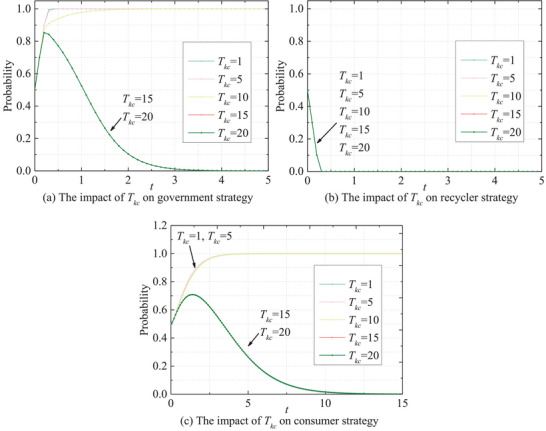
Impact of *T_kc_* on the evolutionary paths of stakeholders' behaviors.

To further investigate the impact of risks on decision‐making, we set the initial probabilities *x*_0_, *y*_0_, *z*_0 _= 0.5 and conduct a univariate evolutionary analysis using risk parameters (*t* = 20). **Figure** **13** shows the evolution path of the stakeholders at different levels of *T_kc_*. The results indicate that the probability of the government adopting active behavior varies significantly with *T_kc_*. When *T_kc_* takes a relatively lower value, the probability quickly approaches 1 and remains stable, suggesting that rapid risk processing strongly encourages active strategies. As *T_kc_* increases, the growth of the probability slows and eventually declines significantly. For *T_kc _* = 15 and 20, the probability peaks early and then decreases continuously, approaching zero over time. This indicates that longer risk handling times discourage active behavior and may lead to a shift toward passive strategies. Obviously, the probability of recyclers adopting active behavior drops rapidly to zero and remains stable across all scenarios, indicating that they consistently tend toward passive behavior. In contrast, consumers' behaviors are significantly influenced by *T_kc_*. This suggests that longer *T_kc_* discourages consumers from choosing active strategies. In summary, *T_kc_* exhibits a negative correlation with the inclination of both government and consumers toward opting for active strategies, while showing no significant impact on the behaviors of recyclers. This disparity may stem from the fact that recyclers base their decisions more heavily on alternative determinants, prioritizing economic gains such as costs and benefits over risk mitigation capacities.

**Figure 14:**
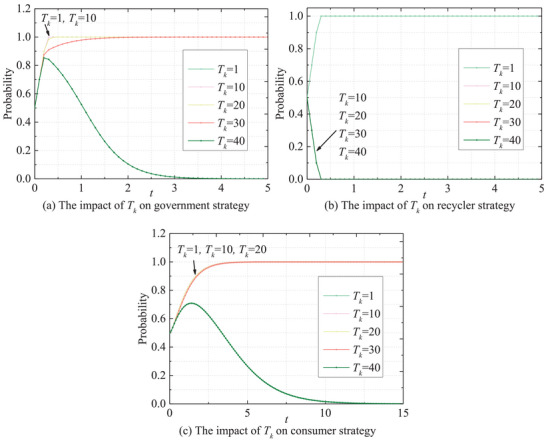
Impact of *T_k_* on the evolutionary paths of stakeholders' behaviors.

Subsequently, the impact of the risk period *T_k_* on the stakeholders' strategy is investigated (see **Figure** **14**). Exactly, when *T_k_* remains at a low level, the probability that the government adopts an active strategy rises rapidly and stabilizes at one. With the increased *T_k_*, the growth rate of the probability of the government adopting active strategies is significantly slowed down, and the convergence rate decreases notably. Recyclers' behavior becomes polarized under the influence of the *T_k_*, they tend to adopt active strategies only when the risk period is extremely short. This indicates that the choice of recyclers' strategies has a critical characteristic; an extremely short risk period could force them to respond actively, but once the risk period exceeds a threshold, the recyclers' behavior becomes completely negative. Therefore, the core motivation of recyclers' decision‐making still remains economic cost‐benefit. While for consumers, they tend to adopt negative strategies when *T_k_* is sufficiently large.

**Figure 15:**
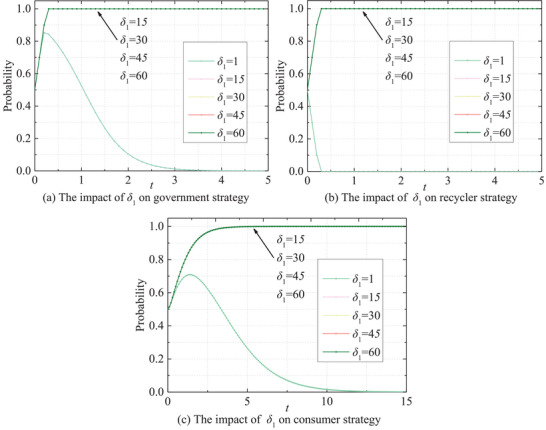
Impact of *δ*_1_ on the evolutionary paths of stakeholders' behaviors.

**Figure 16:**
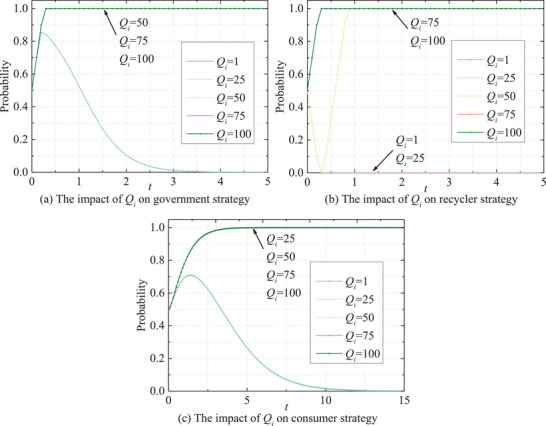
Impact of *Q_i_* on the evolutionary paths of stakeholders' behaviors.

Ultimately, the risk intensity *Q_i_* and penalty coefficient *δ*_1_ exhibit similar impacts on the behavioral dynamics of three parties, as depicted in **Figures** **15** and **16**, exerting significant influence on their behavioral evolution. Governments and recyclers demonstrate sensitivity to variations in *Q_i_* and *δ*_1_. Lower levels of *Q_i_* and *δ*_1_ tend to prompt the adoption of passive strategies by both parties. Conversely, as *Q_i_* and *δ*_1_ escalate, their likelihood of adopting active strategies increases rapidly, approaching unity. This trend underscores how heightened risk intensity strongly incentivizes governments and recyclers to embrace active regulatory approaches in mitigating potential risks. Consumer behavior, in contrast, appears to be less profoundly affected by these two factors. While elevations in *Q_i_* and *δ*_1_ can enhance the growth rate of consumer positivity, the magnitude of this effect is notably smaller compared to that observed in governments and recyclers.

**Figure 17:**
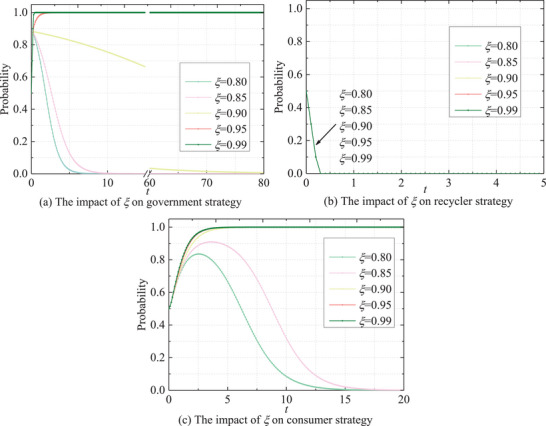
Impact of *ξ* on the evolutionary paths of stakeholders' behaviors.

**Figure** **17** illustrates the evolution of stakeholders' strategies across various confidence levels (***ξ*** = 0.8, 0.85, 0.9, 0.95, 0.99). For ***ξ*** ≤ 0.90, the government tends to shift toward passive strategies following a transient fluctuation. A lower confidence level corresponds to quicker convergence toward passive strategies, indicating that the government is more likely to adopt active strategies when they prioritize financial losses and political risks in highly adverse situations, such as policy failures or public events. In contrast, recyclers consistently favor passive strategies, but with significant incentives, such as increased gains from punitive measures or formal recycling, they transition toward active strategies. Consumers' strategies alignment mirrors that of governments, with higher confidence levels leading to a swifter rise in consumer probability curves, culminating in earlier stabilization. This underscores stakeholders' risk perception as a potent motivator for heightened engagement in recycling activities to mitigate potential penalties or losses associated with non‐compliance.

## 5 Discussion

To better understand the impact of risk on the strategic interaction and evolution path among agents in the retired power battery recovery system, a quantitative risk‐induced loss model is constructed by combining network calculations with CVaR. Specifically, this paper analyzes the impact of risk parameters on stakeholder income and risk‐induced loss, and then further explores how these risk parameters affect the evolution of stakeholder decision‐making.

Returning to question one posed in the introduction, we clarify the impact of risk parameters on revenues through sensitivity analysis. We examined the specific impacts of various risk parameters on revenues. The results show that the impacts of risk parameters on government and recyclers' revenues are contingent on the employed strategy. Specifically, the subject exhibits heightened sensitivity to risk under the negative strategy. Consumers' revenues, on the other hand, are not directly affected by risk but are indirectly influenced by the strategic decisions of the government and recyclers. Conversely, recyclers encounter a multitude of risk factors in cases of non‐compliance behavior. Over time, these risks proliferate, diffuse, and accumulate, leading to a substantial decline in revenue. Therefore, timely intervention to mitigate risks is imperative to curb their proliferation and avert significant losses. These observations align with existing literature.^[^ ^36^, ^41^ ^]^ Moreover, our findings, grounded in a theoretical framework of risk, make a valuable contribution to the body of knowledge on risk management in battery recycling.

To address question two, we conducted orthogonal experiments using the Taguchi technique to assess the impact of risk parameters on risk‐induced loss. Specifically, we investigated the optimal levels and contributions of various risk parameters to risk‐induced losses. Our findings revealed distinct temporal characteristics in the effects of different risk factors on the system. Initially, risk management capacity exerted a prominent influence, albeit diminishing rapidly, indicating transient behavior. Conversely, risk intensity, risk period, and penalty coefficient exhibited cumulative and delayed effects, with risk intensity emerging as the primary driver of risk‐induced loss persistence. The system displayed notable volatility in its initial phase, underscoring the need for heightened vigilance in risk management during the system's inception. These observations underscore the critical role of risk management in battery recycling systems.

Finally, to answer question three, we use risk parameters to perform univariate evolutionary game analysis to explore their impact on coevolutionary systems. The simulation results reveal substantial variability in the impact of risk on third‐party decision‐making behavior. Additionally, government decision‐making exhibits volatility when risks are low, which is similar to that observed in Nie's study,^[^ ^38^ ^]^ resulting in fluctuations in consumer decision‐making. The findings indicate that risk and government penalties exert similar influences, albeit in contrasting manners, to the positive guidance provided by the government to recyclers and consumers through subsidies and incentives. When the risk‐induced loss is low, stakeholders tend to adopt negative strategies. As the risk increases, stakeholders are compelled to reassess the drawbacks of negative strategies, leading to a gradual rise in the likelihood of adopting positive strategies. Thus, it is posited that appropriate risk levels can facilitate the sustainable development of the recycling market to a certain extent. These risks encompass technical, economic, managerial, personnel, and environmental dimensions, permeating all facets of the recycling system. Risk serves as a catalyst for upgrading recycling technologies, enhancing management practices, training personnel, and prioritizing environmental conservation. While not all approaches aim to eradicate risk, they aim to find a harmonious equilibrium between risk and benefit. Ultimately, this fosters the efficient recycling of batteries, aligning with prior research findings.^[^ ^36^, ^41^ ^]^

The implementation of policy effects requires time.^[^ ^14^ ^]^ Consumers, as providers of retired batteries, only indirectly participate in battery recycling management activities; they exhibit a lack of concern for the associated risks. Therefore, the decision‐making of consumers is primarily affected by government incentives and the potential profitability derived from different recycling channels. Consumers' responses to risk are limited in intensity of response due to the time required for policy effects to materialize. In high‐risk contexts, effective government intervention and prompt action by recyclers are crucial for upholding system stability. Risk permeates the entire recycling network, exerting direct or indirect influence on all stakeholders. Our research highlights variations in strategy decisions made by government, recyclers, and consumers considering risks. These disparities offer a novel foundation for investigating stakeholders' inclination toward active participation. Our findings on the heterogeneous risk responses among government, recyclers, and consumers are consistent with the multi‐stakeholder perspectives identified by Bhuyan et al.,^[^ ^46^ ^]^ who highlight divergent priorities and risk perceptions across different actor groups in the battery recycling ecosystem.

## 6 Conclusion

In conclusion, this work analyzes the evolutionary game dynamics among government, recyclers, and consumers from a risk perspective. Numerical simulations are employed to investigate the effects of risk parameters on stakeholders' revenues and risk‐induced losses, and to analyze the impact mechanism of risk on stakeholders' behavior evolution. The findings of the research are concluded as follows:

1)The impact of risk on government and recyclers is strategy‐dependent, with both government and recyclers exhibiting heightened sensitivity to risk under negative strategies. It is imperative for both government and recyclers to prioritize risk management during the battery recycling process to mitigate potential substantial losses or public safety hazards stemming from inadequate risk control measures.
In the early stage of risk occurrence, the main factors influencing risk‐induced loss are sequentially risk management capability, penalty coefficient, risk intensity, and risk period, all of which serve as key regulatory variables for risk‐induced loss. And the impact of these risk parameters dynamically changes with the risk occurrence time. Recyclers need to improve their risk management capabilities and increase the flexibility of their recycling networks.
Government decision‐making can display volatility when risks are low, leading to fluctuations in consumer decision‐making. The government needs to adapt policies in response to market conditions to effectively guide outcomes.
Risks act as a reverse pushback to promote the sustainable development of the recycling market. The government and recyclers may need to weigh the revenues and risks of recycling.

Finally, there are some limitations in the research methodology. We currently overlook volatility and extreme events by relying on the average probability of risk occurrence, indicating a need for future enhancements in this area. Moreover, investigating the interplay of multiple key factors, adapting the recovery strategy dynamically, and examining the effects of dynamic risk intensity and node heterogeneity on evolutionary trajectories represent promising avenues for future research. Lastly, delving into how subsidy mechanisms interact with various risk types and collectively influence system evolution presents a valuable direction for further exploration.

## Conflict of Interest

The authors declare no conflict of interest.

## Data Availability

The data that support the findings of this study are available from the corresponding author upon reasonable request.
